# Supplementary material for: A UMLS-based spell checker for natural language processing in vaccine safety
Source: BMC Med Inform Decis Mak. 2007 Feb 12;7:3. doi: 10.1186/1472-6947-7-3 (PMC1805499; doi:10.1186/1472-6947-7-3)
Supplement: Additional file 13 — RAP application directory. Sets up directory for RAP (RDF application for PHP) source code [file 1472-6947-7-3-S13.gz › rap/doc/phpdoc/ontModel/RdfsVocabulary.html]

xml version="1.0" encoding="iso-8859-1"?


Docs For Class RdfsVocabulary


## Class RdfsVocabulary

Description

Description |
Methods (details)

RDFS vocabulary items

History: <LI>10-05-2004 : First version of this class.</LI>

- access: public
- author: Daniel Westphal <at d-westphal dot de mail at d-westphal dot de>
- version: V0.9.1

Located in /ontModel/RdfsVocabulary.php (line 23)

```
OntVocabulary
   |
   --RdfsVocabulary
```

Method Summary

Description |
Methods (details)

object ResProperty
COMMENT
()

object ResProperty
DOMAIN
()

object ResProperty
IS\_DEFINED\_BY
()

object ResProperty
LABEL
()

string
NAMESPACE
()

object ResResource
ONTCLASS
()

object ResProperty
RANGE
()

object ResProperty
SEE\_ALSO
()

object ResProperty
SUB\_CLASS\_OF
()

object ResProperty
SUB\_PROPERTY\_OF
()

object ResProperty
TYPE
()

Methods

Description |
Methods (details)

COMMENT (line 55)

Answer the predicate that denotes comment annotation on an ontology element.

- access: public

object ResProperty

COMMENT
()

DOMAIN (line 43)

Answer the predicate that denotes the domain of a property.

- access: public

object ResProperty

DOMAIN
()

IS\_DEFINED\_BY (line 66)

Answer the predicate that denotes isDefinedBy annotation on an ontology element

- access: public

object ResProperty

IS\_DEFINED\_BY
()

LABEL (line 77)

Answer the predicate that denotes label annotation on an ontology element

- access: public

object ResProperty

LABEL
()

NAMESPACE (line 132)

Answer the string that is the namespace prefix for this vocabulary

- access: public

string

NAMESPACE
()

ONTCLASS (line 32)

Answer the resource that represents the class 'class' in this vocabulary.

- access: public

object ResResource

ONTCLASS
()

RANGE (line 88)

Answer the predicate that denotes the domain of a property.

- access: public

object ResProperty

RANGE
()

SEE\_ALSO (line 99)

Answer the predicate that denotes seeAlso annotation on an ontology element

- access: public

object ResProperty

SEE\_ALSO
()

SUB\_CLASS\_OF (line 110)

Answer the predicate that denotes that one class is a sub-class of another.

- access: public

object ResProperty

SUB\_CLASS\_OF
()

SUB\_PROPERTY\_OF (line 121)

Answer the predicate that denotes that one property is a sub-property of another.

- access: public

object ResProperty

SUB\_PROPERTY\_OF
()

TYPE (line 143)

Answer the predicate that denotes the rdf:type property.

- access: public

object ResProperty

TYPE
()

Documentation generated on Thu, 7 Jul 2005 13:43:04 +0200 by phpDocumentor 1.3.0RC3
